# Methylmalonic Acidemia: Diagnosis and Neuroimaging Findings of This Neurometabolic Disorder (An Iranian Pediatric Case Series)

**Published:** 2013

**Authors:** Parvaneh KARIMZADEH, Narjes JAFARI, Farzad AHMAD ABADI, Sayena JABBEDARI, Mohammad-Mahdi TAGHDIRI, Hamid NEMATI, Sasan SAKET, Seyed-fakhreddin SHARIATMADARI, Mohammad-Reza ALAEE, Mohammad GHOFRANI, Seyed Hasan TONEKABONI

**Affiliations:** 1 Pediatric Neurologist, Pediatric Neurology Research Center, Shahid Beheshti University of Medical Sciences, Tehran, Iran; 2 Pediatric Neurology Department, Mofid Children Hospital, Faculty of Medicin, Shahid Beheshti University of Medical Sciences, Tehran, Iran; 3 Department of Pediatric Endocrinology, Faculty of Medicin, Shahid Beheshti University of Medical Sciences, Tehran, Iran

**Keywords:** Methylmalonic academia, Neurometabolic disorder, Developmental delay, Early detection

## Abstract

**Objective:**

Methylmalonic acidemia is one of the inborn errors of metabolism resulting in the accumulation of acylcarnitine in blood and increased urinary methylmalonic acid excretion. This disorder can have symptoms, such as neurological and gastrointestinal manifestations, lethargy, and anorexia.

**Materials & Methods:**

The patients who were diagnosed as methylmalonic acidemia in the Neurology Department of Mofid Children’s Hospital in Tehran, Iran, between 2002 and 2012 were included in our study. The disorder was confirmed by clinical findings, neuroimaging findings, and neurometabolic and genetic assessment in reference laboratory in Germany. We assessed the age, gender, past medical history, developmental status, clinical manifestations, and neuroimaging findings of 20 patients with methylmalonic acidemia.

**Results:**

Eighty percent of the patients were offspring of consanguineous marriages. Half of the patients had Failure to thrive (FTT) due to anorexia; 85% had history of developmental delay or regression, and 20% had refractory seizure, which all of them were controlled. The patients with methylmalonic acidemia were followed for approximately 5 years and the follow-up showed that the patients with early diagnosis had a more favorable clinical response in growth index, refractory seizure, anorexia, and neurodevelopmental delay. Neuroimaging findings included brain atrophy, basal ganglia involvement (often in putamen), and periventricular leukomalacia.

**Conclusion:**

According to the results of this study, we suggest that early assessment and diagnosis have an important role in the prevention of disease progression and clinical signs.

## Introduction

Methylmalonic acidemia is a rare inborn neurometabolic disorder with error in aminoacid metabolism (1). This disorder is caused by decrease in activity of methylmalonyl-coA mutase. This enzyme is necessary for catabolism of four amino acid (isoleucine, methionine, threonine, valine) (2,3). Methylmalonic acidemia can present with neurologic deficits, metabolic acidosis, vomiting, lethargy, anorexia, respiratory distress, severe ketoacidosis, and hypotonia. If this disorder is not treated immediately, the patient may go into coma and die (4-6). Increased organic acid level, such as methylmalonic acid may be toxic and harmful for all types of body cells (7). Organs involvement in methylmalonic acidemia includes central nervous system (CNS), bone marrow and kidneys (8). In this study, we present 10 years of experience about methylmalonic academia in the Pediatric Neurology Research Center of Mofid Children’s Hospital, Tehran, Iran. We describe clinical symptoms and neuroimaging findings of 20 cases with this disorder.

## Materials & Methods

This observational study was performed on patients who were diagnosed as methylmalonic acidemia in the Neurology Department of Mofid Children’s Hospital in Tehran, Iran, between 2002. The diagnosis was performed based on clinical manifestations, neuroimaging findings, and finally laboratory assessment of increased acylcarnitine level in blood and methylmalonic acid level detection in urine at metabolic disorders laboratory in Germany. The data of the patients were collected as age, gender, past medical history, developmental status, general appearance, and clinical and neuroimaging findings.

Treatment consisted of cobalamin and carnitine supplements and a low-protein diet. The children’s diet was carefully controlled. If supplements did not help, the doctor could also recommend a diet that avoids, substances such as isoleucine, threonine, methionine, and valine.

The data were analyzed by descriptive method and no statistical testing was applied.

Institutional ethical approval for the conduct of this study was obtained from the Pediatric Neurology Research Center of Shahid Beheshti University of Medical Sciences, Tehran, Iran. All parents signed a written consent for participation in the study.

## Results

Twenty patients with Methylmalonic acidemia were included in this study. They were 10 males and 10 females and their age range was from 3.5 months to 15.6 months. The earliest case was diagnosed in a patient who was 2 months old and the latest case was diagnosed in a 2 year-old patient. The average age of patients at detection time was 31.5 months. Twenty-five percent of the patients had a history of neonatal hospitalization because of prematurity (10% of patients) and icter (15% of patients). In developmental assessment, 50% of patients had developmental delay, 15% had normal development, but 20% of these patients showed developmental delay and regression due to stress, such as infection and fever; and 15% of patients had a normal developmental assessment. In the patients who had a normal developmental assessment, the head circumference and neuroimaging were normal because our survey were done on siblings of our patients with methylmalonic acidemia. Thirty-six percent of the patients had a history of recurrent vomiting and narcosis attack. One of the patients had a complaint of halitosis and undescended testis (UDT). Forty-five percent of patients had a dysmorphic face with protruding forehead and depressed nasal bridge; one patient had polydactyly; three patients had maculopapular lesions and thin hair; and one patient had hepatomegaly. Eightypercentofpatientswereoffspringofconsanguineous marriages, so that in 50% of patients, their parents were first cousins and in 15%, their parents were second cousins, 15% of patients’ parents were family relatives, not first or second cousin. Thirty-six percent of patients had a family history of seizure, developmental delay, or death in their children. Weight in 50% of patients was below the 3% percentile, and height in 10% of patients was below the 5% percentile.

Forty-five percent of patients had microcephaly, 10% had macrocephaly, and 45 % had normal head circumference. Fundoscopic examination in all patients was normal, but 10% of patients had intermittent gaze to one side. Twenty-five percent of patients had spastic; 30% had hypotonicity; 20% had brisk DTR; 60% had a history of seizure (30% tonic, 20% GTC, and 10% infantile spasm);

20% had refractory seizure, that all types of refractory seizure were controlled after diagnosis and treatment of methylmalonic academia; and 20% of patients had agitation and irritability. No abnormality was observed in other physical examinations (chest and abdomen).

In lab data, 50% of patients had decreased levels of hemoglobin, 33% had cytopenia; 15 % had increased levels of ammonia, 20% had increased levels of lactate, and 25% had metabolic acidosis, and one patient had nephritis and proteinuria, and one another patient had chronic renal failure with increased levels of Cr. In addition to methylmalonic academia, three patients had propionic acidemia and one patient had homocystinuria. Electroencephalography (EEG) in 3 patients showed BURST suppression. In neuroimaging data, 50% of patients had brain atrophy, 15% had basal ganglia involvement (mostly in putamen), 10% had periventricular leukomalacia (PVL), and 30% had normal brain MRI.

## Discussion

Methylmalonic acidemia is a neurometabolic disorders with autosomal recessive inheritance, which is caused by methylmalonyl-CoA mutase deficiency. Twenty patients (10 males and 10 females) with methylmalonic acidemia were included in this study. Twenty-five percent of patients had a history of hospitalization because of icter or prematurity. Eighty percent of cases had consanguineous parents, therefore, in suspected cases of methylmalonic acidemia, having consanguineous parents can contribute to the diagnosis. Half of the patients had FTT (weight bellow 3% percentile) because of anorexia, that percentiles of weight and height were improved after the treatment of methylmalonic acidemia. Forty-five percent of patients had microcephaly, 10% had macrocephaly, and 45% had normal head circumference; therefore, the macrocephaly and microcephaly and normal head circumference have not any profit and loss in the diagnosis. Three patients had skin lesions and thin hair, so that their appearance was similar to that of biotinidase-deficient patients. Eight-five percent of patients had a history of developmental delay and regression and evolution only in 15% of patients was normal. Twenty percent of patients had a history of sever agitation and 60% of them had a history of seizure (30% tonic, 20% GTC, and 10% infantile spasm). Twenty percent of the patients had refractory seizure, that all types of refractory seizure were controlled after diagnosis and treatment of methylmalonic acidemia. Seizure detection in our study was more than previous studies. Ma et al. reported seizure in 42.9% of their patients (10). The reason of our high reports of seizure detection, especially infantile spasm, probably is our attention to non-specific presentation and features of seizure (especially infantile spasm). Our patients with methylmalonic acidemia and seizure came to our specialist center and exact evaluations were done about them. Another reason for this difference in the percentage of reported seizure may be related to high frequency of consanguineous marriage between the patients’ parents in our study.

In all our patients with normal development, head circumference, and neuroimaging were normal. The patients were the siblings with methylmalonic acidemia that our survey was done on them in order to prevention of neurological symptoms and signs, early diagnosis, and treatment. Fifty percent of patients had brain atrophy, 15% had basal ganglia involvement (mostly in putamen), 10% had PVL (periventricular leukomalacia), and 30% had normal brain MRI. In 3 patients, EEG showed BURST suppression. In Brismar et al.’s study, mild to moderate myelination delay and basal ganglia changes, especially in globus pallidus were reported (11). In a study by Nicolaides et al. there was reported that seizure, hypotonicity, movement disorders, spasticity, developmental delay, and mental retardation were the manifestations of methylmalonic academia (12).

In a study by Radmanesh et al. on 52 patients (71.2% male and 28.8% female) with methylmalonic academia, cortical atrophy and ventricular dilation were observed in their neuroimaging. The first presentation of their patients were seizure, developmental delay, hypotonicity, and respiratory and gastrointestinal symptoms (3).

**In conclusions,** twenty-five percent of our patients had a history of hospitalization at birth. Eighty percent of patients were offspring of consanguineous marriage, which is an important point to be considered in patients with suspected neurometabolic disorders. There were 3 patients with cutaneous manifestations and thin hair who were similar to patients with other neurometabolic disorders. The incidence of neurological disorders such as developmental delay and regression was found in 85% of patients and seizure was seen in 60% of patients, which this incidence is more than that of other studies. Most patients with normal development, head circumference, and neuroimaging were siblings of methylmalonic acidemia patients that had been analyzed in term of methylmalonic acidemia. Twenty percent of patients had refractory seizure, that all types of seizure were controlled after the treatment of methylmalonic acidemia. 

## Notes

**Declaration of** **conflicting** **interests**

None declared.

**Funding**

The authors received no financial support for the research and publication of this article.
